# Resistance training presents beneficial effects on bone development of adolescents engaged in swimming but not in impact sports: ABCD Growth Study

**DOI:** 10.1186/s12887-024-04634-0

**Published:** 2024-04-09

**Authors:** Ricardo R. Agostinete, André O. Werneck, Pedro H. Narciso, Esther Ubago-Guisado, Manuel J. Coelho-e-Silva, Renata M. Bielemann, Luis Alberto Gobbo, Bruna Turi Lynch, Romulo Araújo Fernandes, Dimitris Vlachopoulos

**Affiliations:** 1 Laboratory of Investigation in Exercise (LIVE), Department of Physical Education, Sao Paulo State University (UNESP), Presidente Prudente, Brazil; 2 Center for Epidemiological Research in Nutrition and Health, Department of Nutrition, School of Public Health, University of São Paulo (USP), São Paulo, Brazil; 3 Department of Physical Education and Sports, Faculty of Sport Sciences, Sport and Health University Research Institute (iMUDS), University of Granada, Granada, Spain; 4 Faculty of Sport Sciences and Physical Education, University of Coimbra, Coimbra, Portugal; 5 Post-Graduate Program in Nutrition and Foods, Federal University of Pelotas, Pelotas, Brazil; 6 Post-Graduate Program in Epidemiology, Federal University of Pelotas, Pelotas, Brazil; 7 Skeletal Muscle Assessment Laboratory (LABSIM), Department of Physical Education, School of Technology and Sciences, São Paulo State University (UNESP), Presidente Prudente, Brazil; 8 Department of Physical Education and Exercise Science, Lander University, Greenwood, SC USA; 9 Children’s Health and Exercise Research Centre, Public Health and Sport Sciences, University of Exeter, Exeter, UK

**Keywords:** Bone mineral density, Adolescence, Sport participation, Physical activity, Bone tissue

## Abstract

**Background:**

Sports practice during adolescence is important to enhance bone development, although it may provide different effects depending on the mechanical impact present in the sport. Besides, resistance training (RT) may also induce bone changes directly (via muscle contractions) and indirectly (via myokines). However, there have been no studies analyzing the longitudinal influence of engaging in sport with and without added mechanical load. Thus, this study aims to analyze the combined effects of sports participation and resistance training on areal bone mineral density (aBMD) accrual in adolescent athletes participating in swimming and impact sports for 12-months.

**Methods:**

This was a 12-month longitudinal study. The sample comprised 91 adolescents (21 females) aged 10 to 18 years, engaged in impact sports (basketball, tennis, track & field, baseball and gymnastics, *n* = 66) and non-impact sport (swimming, *n* = 25). The sample was divided according to resistance training participation: impact sports only (*n* = 45), impact sports + resistance training (*n* = 21), swimming-only (*n* = 17) and swimming + resistance training (*n* = 8). aBMD and soft tissues were measured using dual-energy X-ray absorptiometry. Generalized linear models analysis was used for the resistance training (RT) x type of sport interaction in predicting aBMD changes overtime, adjusting for maturation, sex and baseline aBMD.

**Results:**

After 12-months, all groups showed a significant increase in aBMD, except for the swimming groups (regardless of resistant training), which showed a significant loss in spine aBMD (-0.045 [-0.085 to -0.004] g/cm^2^ in swimming-only and − 0.047 [-0.073 to -0.021] g/cm^2^ in swimming + RT). In comparisons between groups, only swimming + RT group, compared with swimming-only group presented higher upper limbs aBMD (0.096 g/cm^2^ [0.074 to 0.118] in swimming + RT vs. 0.046 [0.032 to 0.060] g/cm^2^ in swimming only; *p* < 0.05) and whole body less head (WBLH) aBMD (0.039 [0.024 to 0.054] g/cm2 in swimming + RT vs. 0.017 [0.007 to 0.027] g/cm^2^ swimming-only; *p* < 0.05).

**Conclusion:**

Despite the significant gain in aBMD in all groups and body sites after 12-months, except for the spine site of swimmers, the results indicate that participation in RT seems to improve aBMD accrual in swimmers at the upper limbs and WBLH.

**Supplementary Information:**

The online version contains supplementary material available at 10.1186/s12887-024-04634-0.

## Introduction

Paediatric sport participation has gained growing attention, mainly because it is a behavior widely spread among children and adolescents [1, 2]. In the American continent, the engagement in different sports ranges from 1.2 to 39.0% in children and 10.2–30.6% in adolescents, while in Europe ranges from 2.4 to 28.5% in children and 3.8–29.0% in adolescents [3]. In fact, sports participation is considered the most common manifestation of physical exercise among children and adolescents [3].

The importance of sports participation during adolescence is enhanced by the fact that regular engagement in sports is also linked to several health benefits, including bone tissue adaptations [4]. Sports involving mechanical load stimulus have a positive effect on bone health due to high ground-reaction and dynamic forces applied to the skeleton. This occurs because mechanical loading damages the tissue and induces bone multicellular units (BMUs) (i.e., osteoclast and osteoblasts) to start a process called bone remodeling to make that region damaged more resistant [5]. In this process, osteoclasts are recruited to remove the damaged tissue and they will also interact with the reversal cells, that initially show a catabolic activity, but when they increase in population and density, they start presenting an osteogenic potential due to its anabolic effect [6]. These stimuli are considered crucial for an ideal peak bone mass and, consequently, to reduce the risk of developing osteoporosis [7].

In contrast, sports performed in low-gravity conditions (e.g. cycling and swimming) have absence of mechanical stimulation and might not be able to produce osteogenic benefits of the same magnitude in aBMD and bone mineral content (BMC) of whole body and body segments [8–10]. Consequently, adolescent athletes engaged in this type of sport lacking mechanical impact tend to present BMD values equal to their non-active peers [8].

Resistance training (RT) is another relevant manifestation of physical exercise in adolescents. Among athletes, RT has an important role in increase in muscle strength and muscle endurance [11, 12], improvements of motor skills [13], and reduction in the risk of injury in adolescent athletes [14, 15]. Therefore, the RT has been a beneficial addition for many adolescents engaged in organized sports [16]. Regarding bone tissue, RT may also enhance bone development of adolescents. Direct and indirect pathways are involved in this association, as muscle contraction strains the bone tissue via tendons (i.e., direct effect) [17], and releases several myokines that interacts with bone cells stimulating bone turnover (i.e., indirect effect, mediated by IGF-1, FGF-2 & 21, irisin) [18].

Therefore, RT could improve bone health of adolescent practitioners of sports performed in low-gravity, such as swimming and even boost the effects of impact sports. Previous studies have sought to understand the isolated effect of different protocols of RT on bone health of adolescents showing its beneficial effect on aBMD accrual [19, 20]. While other studies available in the literature have analyzed the joint effects of sports, such as swimming, when added to impact sports [21] or different interventions as plyometrics [22] and whole-body vibration on bone health [23].

Although there are studies analyzing the isolated impact of RT on bone development among adolescents, as well as other exercise interventions added to sport, there have been no studies analyzing the longitudinal influence of engaging in sport with and without mechanical load added to resistance training (RT) on bone development at different skeletal sites. Thus, the aim of the study was to analyze the association of combined participation in sports (swimming and impact sports) and RT with aBMD accrual among adolescents compared with adolescents who performed the sport singly. We hypothesize that adolescents engaged in sports modalities combined with resistance training will show a greater accumulation of bone mass compared to adolescents who perform the sport singly. However, we posit that this gain may be determined by the type of sports modality practiced, with or without mechanical load.

## Methods

### Sample

This is a longitudinal study that presents findings from 1-year follow-up from adolescents participating in the study “Analysis of Behaviors of Children During Growth” (ABCD Growth Study), carried since 2017 in the city of Presidente Prudente, Sao Paulo state, Brazil. The data used in this manuscript have been collected between 2016/2017 (baseline measures) and 2017/2018. All data collection and analyzes were performed by a trained staff of the Laboratory of Investigation in Exercise (LIVE). The ethics committee of the Sao Paulo State University (UNESP) approved the study (process number 1.677.938/2016). All parents and adolescents signed a written consent form.

Before contacting the potential participants, the researchers asked permission to the local authorities to contact the facilities (schools and sport clubs) and, after the permission was granted, principals and/or coaches were contacted in order to ask permission to contact the adolescents at schools and sport clubs, respectively. In the 11 facilities in which the access was granted to the researchers, adolescents were contacted aiming to explain the aims, benefits and potential risks related to the participation in the ABCD – Growth Study. At baseline, to be eligible, the adolescents should be aged between 10 and 18 years at baseline and do not use regular medication that could affect bone metabolism. Third, written consent form signed by parents and/or legal guardians.

Given the absence of studies in the literature that analyze the combined effects of swimming and resistance training, the minimum sample size needed to detect an effect size of d = 0.95, it was based in total body less head aBMD comparisons between male swimmers and control group [24]. Thus, with a power of 80% and alpha of 5% it would be necessary 15 adolescents per group.

At baseline of ABCD-Growth Study, 285 adolescents of both sexes were contacted, 132 adolescents specifically from impact sports clubs and swimming team which were considered in this study. After 12 months of follow-up 91 adolescents sport practitioners completed the follow-up measures. Dropouts (*n* = 41) were due to adolescents interrupting sports participation, transfer to another squad in a different city or no desire to participate to the follow-up. Thus, final sample was composed by 91 adolescents (21 females) from two groups (impact sports and swimming).

### Sports participation and resistance training

The impact sports group (*n* = 66) included baseball (*n* = 10), gymnastics (*n* = 10), tennis (*n* = 15), basketball (*n* = 23) and track & field (*n* = 8) while the non-impact group included swimming (*n* = 25). Adolescents were divided into four sub-groups according to RT participation: impact sport only (*n* = 45), impact sport + RT (*n* = 21), swimming only (*n* = 17) and swimming + RT (*n* = 8).

Engagement in RT was assessed through a face-to-face interview conducted by the researchers by asking the following questions in baseline measurement: “Are you engaged in RT?” and a dichotomic answer received (yes/no). If yes, the adolescents were asked “How long have you been practicing RT?” and “how many days a week?” so the engagement was used to categorize the groups.

### Somatic maturation

The body mass was measured using an electronic scale (Filizzola PL 150, model Filizzola Ltda, Brazil with a precision of 0.1 kg). Stature and sitting-height were measured using a stadiometer (Sanny, model American Medical of the Brazil Ltda, Brazil, accurate to 0.1 cm) that permitted an estimate of leg length. These anthropometric measurements (body mass, stature, sitting height and leg length) and chronological age were used to calculate the maturity offset (PHV) through mathematical formulas predicted by Mirwald et al. 2002. [25]. This measure denotes the time (years) from/to age at peak height velocity (APHV).

### Areal bone mineral density (aBMD), lean mass (LM) and fat mass (FM)

aBMD (g/cm²), lean mass (LM, kg) and fat mass (%) were measured in a temperature-controlled room using dual-energy x-ray absorptiometry-DXA (models Lunar DPX-NT and Lunar Prodigy advance; General Electric Healthcare, Little Chalfont, Buckinghamshire, UK) with GE Medical System Lunar software. A trained researcher performed all scan and tested the scanner quality before the first exam of each day. The scans were performed using a standardized protocol with the participants remaining in the supine position and wearing only light clothing. Regional analysis for aBMD of upper limbs, lower limbs, spine, and total body less head (TBLH) occurred off-line after the scans took place, setting the lines (rois) as requested for the General Electric Healthcare company and stated in previous studies [26].

### Statistical analysis

Descriptive statistics was composed of mean values, standard deviation (SD) and 95% confidence intervals (95%CI). Crude comparisons between groups were analysed through the independent sample t-test and Mann-Whitney test. Lasty, all the comparisons of aBMD accrual between groups considering the engagement in RT were performed using Generalized Linear Models (GLM), in which the comparisons were controlled by the confounders (maturity offset [PHV], sex, aBMD at baseline of each segment). In comparisons of bone accrual within groups over 12 months, considering the practice of resistance training (RT), Cohen’s d was calculated as the effect size interpretation [27, 28]. The calculation involved using the estimated mean and standard deviation (SD) of each group, with SD determined by the equation: SD = SEM (standard error of the mean) x $\sqrt{n}$ [29]. The effect size was interpreted as: 0.2 small, 0.5 medium, and 0.8 large [30]. Lastly, the estimated means from GLM model (sport alone vs. sport + RT) were also used to analyse bone accrual within groups over 12-months by trend analysis (considering the confidence interval of each group). All data analysis was conducted using the software SPSS (version 23.0).

## Results

The descriptive characteristics of the sample are presented in Table 1. About the training parameters in each sport group, impact sports + RT presented similar frequency of training, volume of training (per day), week volume of training and time of engagement in the sport (in months) compared with impact sports-only. Similar results were observed in comparisons between swimming + RT group and swimming-only, except for time of engagement. About RT parameters, impact sport group showed a mean of 9.3 ± 15.7 vs. 22.0 ± 13.6 in swimmers of time of engagement (in months) and about week training frequency (days/week) impact sport had 2.4 ± 1.0 vs. 2.8 ± 1.5 in swimmers.

**Table 1:** Descriptive characteristics of the sample stratified by sports at baseline and resistance training engagement (*n* = 91)

	Resistance Training Engagement					
	Impact sports + RT (*n* = 21)	Impact sport-only (*n* = 45)	p-value	Swimming + RT (*n* = 8)	Swimming-only (*n* = 17)	p-value
Variables-Baseline	Mean (SD)	Mean (SD)		Mean (SD)	Mean (SD)	
Boys / Girls	10 / 11	38/7	---	5 / 3	12 / 5	---
Age (year)	14.7 (1.6)	13.3 (1.6)	0.003	16.1 (1.9)	13.4 (2.2)	0.009
Body weight (kg)	58.3 (14.4)	58.0 (13.3)	0.941	64.8 (9.6)	50.9 (11.4)	0.007
Height (cm)	167.7 (9.0)	168.0 (12.6)	0.935	171.8 (7.7)	158.7 (9.9)	0.003
Body fatness (%)	21.6 (8.3)	20.4 (10.9)	0.322	16.7 (6.8)	22.3 (8.9)	0.131
LST (kg)	43.7 (9.90	42.9 (10.6)	0.767	50.0 (13.3)	36.9 (9.3)	0.011
Maturity offset (year)	1.6 (1.5)	0.2 (1.4)	< 0.001	2.5 (1.4)	0.1 (1.8)	0.003
**Training parameters**						
Frequency of training (days/week)	3.3 (1.9)	3.5 (1.7)	0.674	5.9 (0.4)	5.7 (0.8)	0.842
Training volume (minutes/day)	152.9 (74.6)	162.0 (58.9)	0.678	178.1 (76.2)	142.9 (16.0)	0.215
Weekly training volume (min/week)	590.0 (473.4)	619.3 (407.6)	0.547	1050.0 (468.3)	817.1 (153.9)	0.262
Time of engagement (months)	49.7 (28.8)	40.0 (29.5)	0.095	94.5 (32.0)	57.5 (44.2)	0.023

SD = standard deviation; LST = lean soft tissue

### Bone accrual in each group over 12-months

The accrual of BMD over 1-year within groups is shown in values of adjusted mean of change (g/cm^2^) and significance observed by confidence interval. In upper and lower limbs, all groups significantly increased aBMD: impact sport + RT (0.077 [0.055 to 0.099] g/cm^2^ for upper limbs and 0.066 [0.043 to 0.088] g/cm^2^ for lower limbs); impact sport only (0.060 [0.046 to 0.074] g/cm^2^ for upper limbs and 0.084 [0.069 to 0.098] g/cm^2^ for lower limbs); swimming + RT (0.096 [0.074 to 0.118] g/cm^2^ for upper limbs and 0.054 [0.032 to 0.078] g/cm^2^ for lower limbs); swimming only (0.046 [0.032 to 0.060] g/cm^2^ for upper limbs and 0.060 [0.045 to 0.074] g/cm^2^ for lower limbs).

In terms of spine, adolescents engaged in impact sports (independently of engagement in RT) had significant aBMD accrual (0.039 [0.015 to 0.064] g/cm^2^ in impact sports + RT and 0.033 [0.017 to 0.049] g/cm^2^ in impact sport-only). While swimmer (also independently of engagement in RT) had significant aBMD reduction (-0.045 [-0.085 to -0.004] g/cm^2^ in swimming + RT and − 0.047 [-0.073 to -0.021] g/cm^2^ in swimming only). Finally, in WBLH, all groups showed significant aBMD accrual: impact sport + RT (0.065 [0.047 to 0.082] g/cm^2^); impact sport only (0.059 [0.048 to 0.071] g/cm^2^); swimming + RT (0.039 [0.024 to 0.054] g/cm^2^) and swimming only (0.017 [0.007 to 0.027] g/cm^2^) (Table 2).

**Table 2:** Bone mineral density at baseline, after 12-months and mean of difference within groups according to resistance training participation (*n* = 91)

Variables	Impact sports + RT (*n* = 21)	Impact sports only (*n* = 45)	Swimming + RT (*n* = 8)	Swimming only (*n* = 17)
Crude aBMD (g/cm^2^)	Mean (SD)	Mean (SD)	Mean (SD)	Mean (SD)
**Upper limbs**				
Baseline	0.827 (0.114)	0.798 (0.116)	0.865 (0.066)	0.750 (0.115)
Follow-up	0.901 (0.106)	0.859 (0.103)	0.927 (0.069)	0.812 (0.080)
Mean of change	0.074 (0.055)	0.062 (0.050)	0.062 (0.021)	0.062 (0.052)
Mean of change adjusted#	0.077 (0.055 to 0.099)	0.060 (0.046 to 0.074)	0.096 (0.074 to 0.118)	0.046 (0.032 to 0.060)
**Lower limbs**				
Baseline	1.299 (0.158)	1.259 (0.181)	1.234 (0.119)	1.127 (0.135)
Follow-up	1.352 (0.144)	1.348 (0.178)	1.277 (0.119)	1.192 (0.130)
Mean of change	0.053 (0.043)	0.089 (0.056)	0.043 (0.019)	0.065 (0.036)
Mean of change adjusted#	0.066 (0.043 to 0.088)	0.084 (0.069 to 0.098)	0.054 (0.032 to 0.078)	0.060 (0.045 to 0.074)
**Spine**				
Baseline	1.120 (0.152)	1.019 (0.141)	1.133 (0.082)	0.974 (0.150)
Follow-up	1.148 (0.137)	1.057 (0.137)	1.063 (0.088)	0.939 (0.125)
Mean of change	0.028 (0.064)	0.038 (0.052)	-0.069 (0.062)	-0.035 (0.059)
Mean of change adjusted#	0.039 (0.015 to 0.064)	0.033 (0.017 to 0.049)	-0.045 (-0.085 to -0.004)	-0.047 (-0.073 to -0.021)
**WBLH**				
Baseline	1.077 (0.123)	1.041 (0.134)	1.057 (0.083)	0.951 (0.112)
Follow-up	1.131 (0.114)	1.105 (0.130)	1.072 (0.088)	0.979 (0.085)
Mean of change	0.054 (0.041)	0.064 (0.042)	0.015 (0.018)	0.028 (0.036)
Mean of change adjusted#	0.065 (0.047 to 0.082)	0.059 (0.048 to 0.071)	0.039 (0.024 to 0.054)	0.017 (0.007 to 0.026)

RT = Resistance training; SD = standard deviation; aBMD = areal bone mineral density; # results presented in mean and 95% confidence interval and from GLM model of comparisons between groups (sport alone vs. sport + RT) adjusted by maturity offset, sex, and aBMD of each segment at baseline

### Comparisons of bone accrual between groups over 12-months considering the absence of practice in RT

To verify the isolated influence of participation in sports with and without mechanical load on aBMD accrual, GLM comparative adjusted analyses were conducted between impact sports only and swimming only groups. Impact sports presented higher aBMD accrual compared with swimming at lower limbs (mean of 0.059 [0.034 to 0.084] g/cm2 in swimming + RT and 0.092 [0.077 to 0.106] in impact sports + RT; *p* = 0.035), spine (mean of -0.040 [-0.064 to -0.016] g/cm2 in swimming + RT and 0.040 [0.025 to 0.054] in impact sports + RT; *p* < 0.001) and TBLH (mean of 0.022 [0.038 to 0.041] g/cm2 in swimming + RT and 0.066 [0.056 to 0.077] in impact sports + RT; *p* < 0.001).

### Comparisons of bone accrual within groups over 12-months considering the practice of RT

**Fig. 1:**
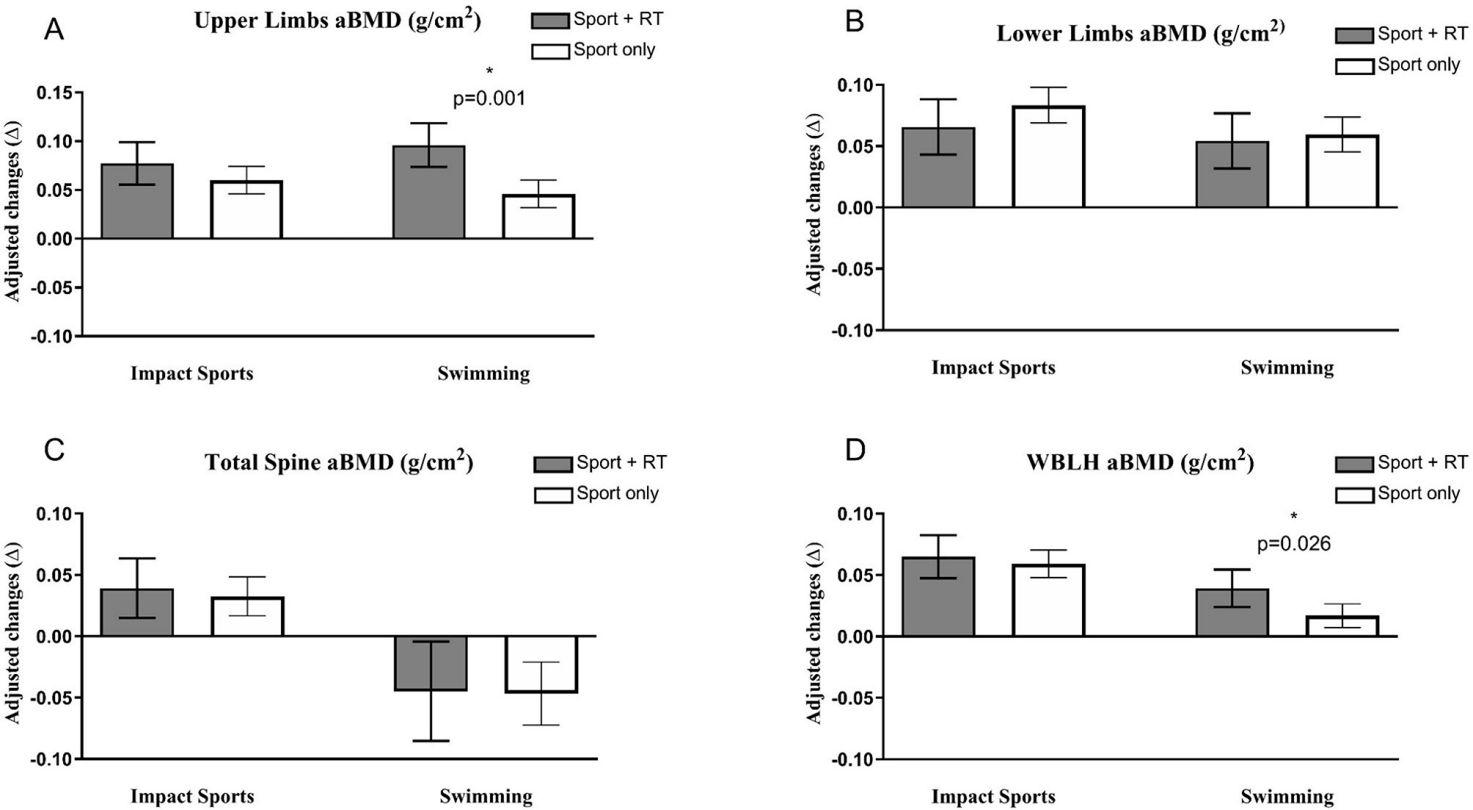
Comparisons of aBMD changes after 12-months by sport and practice of resistance training in upper, spine, lower limbs and whole body adjusted by maturity offset, sex, and aBMD of each segment at baseline (*n* = 91)

The comparations within each group considering engagement in resistance training are shown in values of mean of difference (g/cm^2^). In upper limbs, swimmers + RT presented higher aBMD accrual compared with swimming only (*p* = 0.001; effect size = 1.70[large]) (Fig. 1, Panel A), while impact sport groups presented similar gains (*p* = 0.222; effect size = 0.36 [small]). However, in lower limbs and spine, swimming + RT group showed similar changes in aBMD compared to the swimming-only group (*p* = 0.720; effect size = 0.18 [trivial] in lower limbs and *p* = 0.939; effect size = 0.05 [trivial] in spine;) (Fig. 1, Panel B). Similar results were observed in comparisons between the impact sports + RT and impact sports only (*p* = 0.222; effect size = 0.36 [small] in lower limbs and *p* = 0.674; effect size = 0.12 [trivial] in spine) (Fig. 1, Panel C). Finally, in terms of the TBLH, despite the impact sports group showing similar gains (*p* = 0.617; effect size = 0.15 [trivial]), while swimming + RT presented higher aBMD accrual compared with swimming only (*p* = 0.026; effect size = 1.12 [large]) (Fig. 1, Panel D).

### Comparisons of bone accrual between groups over 12-months considering practice in RT

Lastly, to confirm the potential influence of RT on bone mineral accrual of swimmers, it was performed analyses comparing aBMD accrual between swimming + RT and impact sports + RT. Impact sports + RT presented higher aBMD gain in spine (mean of -0.053 [-0.093 to -0.014] g/cm^2^ in Swimming + RT and 0.022 [-0.001 to 0.045] to in impact sports + RT; *p* = 0.002) and TBLH (mean of 0.022 [0.001 to 0.042] g/cm^2^ in Swimming + RT and 0.052 [0.040 to 0.06] in impact sports + RT; *p* = 0.020).

## Discussion

This longitudinal study aimed to identify the combined impact of engagement in RT and sports participation on aBMD accrual among adolescent athletes of swimming and impact sports. Corroborating the initial hypothesis that the positive effect of resistance training could depend on the sports modality (with or without impact), the main finding of this manuscript hints that engagement in RT seems to boost the aBMD accrual of upper limbs and whole body in swimmers (large effect size), proving to be a good strategy to enhance bone gain in this population. However, these swimmers still present a smaller gain in aBMD in the spine and total body compared to adolescents who practice impact sports in addition to resistance training.

Firstly, our results suggest that, in an analysis of isolated sports practice (without considering engagement in RT), those practicing impact sports showed greater aBMD gains in the lower limbs, spine, and total body compared to adolescents who practiced only swimming. Indeed, previous evidence clearly indicate that swimming seems to be inefficient to promote bone accrual during adolescence mainly in lower limbs because it is a sport performed in a “hypogravity” condition, reducing significantly stimulus related to ground-reaction force and tensions on bone matrix [8, 9]. Even with swimmers presenting high values of muscle mass, the muscle-bone unit may not induce positive adaptations on bone tissue via muscle function in swimmers [31]. Thus, the literature demonstrates that in swimmers, additional osteogenic exercises are necessary to increase BMD values [8], aiming an optimal peak of bone mass and therefore, decreasing the risk of osteoporosis throughout life.

Therefore, recently some studies have sought to understand the effect of different intervention models on bone health of adolescent swimmers as by Gomez-Bruton et al., 2017 [23] and Vlachopoulos et al., 2018 [22]. The study developed by Gomez-bruton et al. [23] analyzed 6 months of whole-body vibration training in bone mass acquisition of adolescent swimmers, concluding that the training protocol was not effective. On the other hand, Vlachopoulos et al. [22], analyzed a 9-month jumping intervention in bone variables of adolescents involved in different sports and concluded that the jumping-intervention is beneficial in improving bone outcomes in non-osteogenic sports, such as swimming and cycling but not football.

RT presents high muscle activity (isometric, concentric and eccentric contractions), which are responsible for bone modeling/remodeling due load applied to skeleton, converting mechanical stimuli to biochemical response by the mechanoreceptors as explained by the “mechanotransduction” and “muscle-bone-unit” theory [17]. These stimuli justify our findings in which higher aBMD accrual on upper limbs and WBLH was observed among swimmers who practice complementary training. Thus, although there are no randomized clinical trial studies analyzing intervention models of RT on athlete adolescents (mainly due to its impact on their training routine), based in our findings, it seems reasonable recommend that RT be integrated to training routine of swimmers.

On the other hand, our findings show that this boost effect of RT on aBMD accrual did not occur in adolescents who practice impact sports. An explanation presented by Vlachopoulos et al. [22] in his findings involving 9-months of jumping intervention, justifies our results. According to the authors, footballers did not show improvement on bone due to a higher threshold for bone improvement due to sport-specific training, which is proven to be beneficial to bone tissue [22, 32]. Another hypothesis pertains to the potential ceiling effect (threshold level) [33], in which adolescents participating in these sports may have already attained the maximum (additional) bone density accumulation that their skeleton can reach through physical strain. In such scenario, the practice of resistance training could not generate additional BMD gains, which would also justify our findings and thus, futures studies should be performed to confirm theses hypotheses.

In contrast, our results reinforce that swimmers seem to be more susceptible to the osteogenic benefits of mechanical stimulates, even when compared to adolescents engaged in osteogenic modalities (e.g. soccer) [34]. In this case, swimming promotes low loads in the bone tissue due to the absence of ground reaction forces, thus the threshold for a remodeling becomes lower than the normal, although more achievable when associated to osteogenic activities [33]. The same principal of a “threshold for a remodeling” seems to be applied to the way body segments are affected by RT, where lower limbs adapted to higher bone strain than upper limbs were less prone to have aBMD accrual boosted by the engagement in RT.

To the best of our knowledge, it was the first study to analyze longitudinally the combination of RT and sports participation on bone health of adolescents. However, limitations should be mentioned. First, the lack of detailed information about the RT is a relevant concern in our study because does not allow to assess training information (e.g. number of sets, periodization, weight lifted) and hence their specific impacts on aBMD. This lack of this information limits our ways to explore potential explanations to findings related to aBMD on spine (e.g. absence of exercises affecting this body region). However, the idea to implement a specific RT intervention in a training routine of athletes competing at national and international level seems of hard implementation.

Second, the study’s sample size did not reach the sample size estimation, and it may affect comparisons between groups. However, it is important to highlight that theoretically, not reaching the desired sample size would be more related to a type 2 error. Therefore, it is expected that no differences between the groups will be observed. However, differences were observed in this study even with a lower sample size (8 in swimming + RT), which would reinforce the hypothesis that this difference exists between them (swimming + RT vs. swimming only). In other way, considering the relative homogeneity of our sample and unmeasured confounding variables that could affect comparisons, these factors could increase the risk of a type 1 error. Thus, future studies with larger sample sizes must be encouraged to confirm the hypotheses.

Besides that, the sample size did not allow to perform analyzes stratified by sex, limiting sex-specific findings. Third, the absence of measures of bone geometry and architecture what limits a deep understanding about how bone adapts in response to combined stimulus of sport and RT. Besides that, it was necessary to use two different DXA equipment. Thus, considering that each DXA has an estimation error, performing analyzes of the same individual on two different equipment could affect the aBMD gain mainly in spine.

Lastly, swimmers practicing RT were older and with a more advanced stage of maturation and for this reason all analyzes were adjusted considering that. Thus, new studies should be encouraged considering analyzes by age groups separately. On the other hand, these differences between the swimming + RT and swimming-only reinforce the osteogenic potential of RT, considering that circumpubertal years (− 2 to + 2 years from PHV) are the period with the greatest accumulation of bone mass during growth (Baxter-Jones et al., 2011), the swimming-only group would be more susceptible to accumulating more bone mass, which did not happen.

## Conclusion

In summary, participation in RT improve the aBMD accrual at upper limbs and WBLH of adolescent swimmers, which may represent an important factor in achieving an optimal peak bone mass. Thus, randomized clinical trials and longer follow-up periods are important to corroborate the findings of this study. Exercise professionals responsible by young swimmers could implement RT in adolescents to promote bone health, in addition to improving performance and reducing injuries. However, the practice of impact sports training, whether alone or added to resistance training, still appears to promote greater bone mass gain.

### Electronic supplementary material

Below is the link to the electronic supplementary material.

Supplementary Material 1

## Data Availability

The research data supporting this publication are available as supplementary information accompanying this publication.
